# Communal roosts of the Blue-fronted Amazons (*Amazona aestiva*) in a large tropical wetland: Are they of different types?

**DOI:** 10.1371/journal.pone.0204824

**Published:** 2018-10-17

**Authors:** Gláucia Helena Fernandes Seixas, Guilherme Mourão

**Affiliations:** 1 Fundação Neotrópica do Brasil, Bonito, Mato Grosso do Sul, Brazil; 2 Embrapa Pantanal, Corumbá, Mato Grosso do Sul, Brazil; Universitat de Barcelona, SPAIN

## Abstract

Psittacidae species are among the most threatened birds in the world. Approximately one-half of the 390 parrot species are experiencing population declines. The Blue-fronted Amazon (*Amazona aestiva*) is the most traded parrot worldwide and suffers from poaching and habitat loss. Many species of parrots, including the Blue-fronted Amazon, form communal roosts where they spend the night. Under certain circumstances, roost surveys can be a rapid and cost-effective way to obtain information about the demography of parrots or the consequences of threats. We surveyed an area of 2,700 km^2^ in a large wetland in mid-western of Brazil and located five Blue-fronted Amazon roosts. We conducted monthly counts of the birds arriving at these roosts for 28–61 months and stratified the counts into flock sizes. We used this information to estimate the number of parrots using these roosts to determine whether the roosts follow seasonal patterns and whether they have different flock-size structures and different dynamics throughout the year, as well as to determine the trends of the roosting parrots, which are stratified by flock size. The roosts were different, as they followed different seasonal patterns and had different flock-size structures, which could be interpreted in relation to the parrot breeding cycle. The trends of singletons, which index the number of reproductive couples each year, and the number of pairs parrots increased or fluctuated around a baseline, but the number of fledged young in the year declined throughout the study. This is of concern, as it indicates problems in population recruitment, which could have been unnoticed by the management authorities, as the total numbers were not decreasing. Although every monitored roost had birds of each age or reproductive condition strata, the fact that the roosts were different could be important in terms of management, as it will be more effective for the conservation of the Blue-fronted Amazon to protect a carefully chosen set of complementary roosts.

## Introduction

The family Psittacidae includes a large proportion of threatened species, and at least one-third of the 390 parrot species worldwide are in some category of risk [1]. Mainly due to trade and habitat loss, more than 55% of the surveyed populations of Neotropical parrots are suffering population declines [2]. The Blue-fronted Amazon (*A*. *aestiva*) is thought to be one of the most frequently traded Psittacidae species worldwide [3, 4] including Brazil [5]. Despite the heavy trade pressure, it is still considered to be at least concern by The International Union for Conservation of Nature (IUCN) [1] and is considered to be an almost-threatened species in Brazil [6], although it was acknowledged that the current population trend is decreasing [1].

Many species of Psittacidae, including the Blue-fronted Amazon, are known to form communal roosts, which are spatially separated from the cavities where they reproduce. The cavities are used by pairs during the reproductive season and are dispersed in the landscape. Communal roosts are composed of a patch of multiple trees where a large number of birds congregate to spend the night and seem to result from their gregarious habits [7, 8]. It is likely that these roosts provide multiple benefits in terms of the exchange of social information [9], feeding efficiency [9, 10] and a reduction of predation risks [11]. Such benefits are not mutually exclusive and may act in a complementary fashion for every roosting parrot [12, 13].

A number of studies have described these communal roosts (e.g., [14, 15, 16]), and some of them aimed to obtain information on the population biology of the parrots [17, 18]. Under certain circumstances, roost surveys can be a rapid and cost-effective way to obtain information about the demography of parrots [19] or about the consequences of threats, such as poaching and habitat loss [20, 21].

The use of a roost can vary over the life cycle of a given individual, such as during the breeding cycle or in accordance with food availability throughout the year (i.e., fruit phenology), in order to minimize its energy expenditure. During the reproductive period, adults need to care for the nest and the brood, which could change the roosting numbers [22, 23, 24]. Some authors working with parrots (*Amazona spp*.) have recognized that one individual of each breeding pair would return to the communal roost to spend the night (e.g., [25, 26, 27]). This behavior is also known to occur in Blue-fronted Amazons from the Pantanal and from the Paraná River Basin. Typically, one individual (presumably the female) of each pair stays within the nest cavity day and night during the incubation period, while the other individual (presumably the male) stays outside during the daytime, providing food and protection, and leaves the area at night (GHFS personal observation). Following the reproductive period, the recruitment of the fledged birds would increase the number of roosting parrots, leading to seasonal variation in the number of parrots in the roosts. Some authorities [18] easily distinguished young and adult Yellow-naped Parrots (*Amazona auropalliata*) and used this feature to determine the number of young in small-flocks in order to estimate the proportion of young in the population. Other authors also used the number of birds arriving at the roosts in small flocks to estimate the young fledged (e.g., [28, 29], this study), even when the young and adults did not differ in coloring, they relied on the behaviour and other traits to distinguish between the young and adult birds.

In some cases, different roost locations can be more suitable for certain strata of the population during a particular time of the year, thus leading to some “specialization” of the roosts [25]. The monitoring of roosts that are persistent over time allows insights to be obtained about population dynamics, such as the population size, the proportion of the population attempting to reproduce every year, recruitment, and population trends [30, 31, 32]. This is especially straightforward for small, isolated and/or endangered populations that use few known roost sites [19], which allows for almost complete population counts. However, even in the case of open and large bird populations that use an unknown number of roosts, it is still feasible to acquire insights into the population.

In this study, we aim to (1) obtain an estimate of the number of Blue-fronted Amazon using the monitored roosts, (2) determine whether the number of birds at the roosts follow seasonal patterns and what patterns these are and (3) propose a method based on the number of singletons roosting during the incubation period to estimate the proportion of the population that attempts to reproduce each year. Additionally, we want to (4) determine whether the roosts are of different types (i.e., of different flock-size structures and having different dynamics throughout the year) and (5) determine the trends in the number of parrots using those roosts, stratified by flock size.

## Materials and methods

### Study area

The Pantanal is a large Neotropical wetland covering approximately 160,000 km^2^ of Brazil, Bolivia and Paraguay. The landscape is characterized by a mosaic of semideciduous forests, savannas and flooded grasslands interspersed with forest patches [33] in addition to manmade pastures and, in some locations, irrigated rice. The climate is marked by a rainy season (Oct-Mar) and a dry season (Apr-Sep).

We surveyed an area of approximately 2,700 km^2^ in the southern Pantanal in Brazil looking for roosts of Blue-fronted Amazon. We relied on the information from locals or triangulated the direction of the long flights of the parrots at sunset, which are usually when the parrots leave their feeding and/or reproduction areas to locate their roosts. We located five large roosts, each being used by many hundred Blue-fronted Amazons and conducted monthly counts of the parrots roosting in these five roosts from July 2004 to July 2009. We found also a sixth roost in the region that sheltered several hundreds of parrots. However, it was located in an inaccessible area and was therefore not regularly monitored. In addition to the roosts mentioned above, it is possible that we had missed some small and infrequently used roosts in the region.

**Fig 1:**
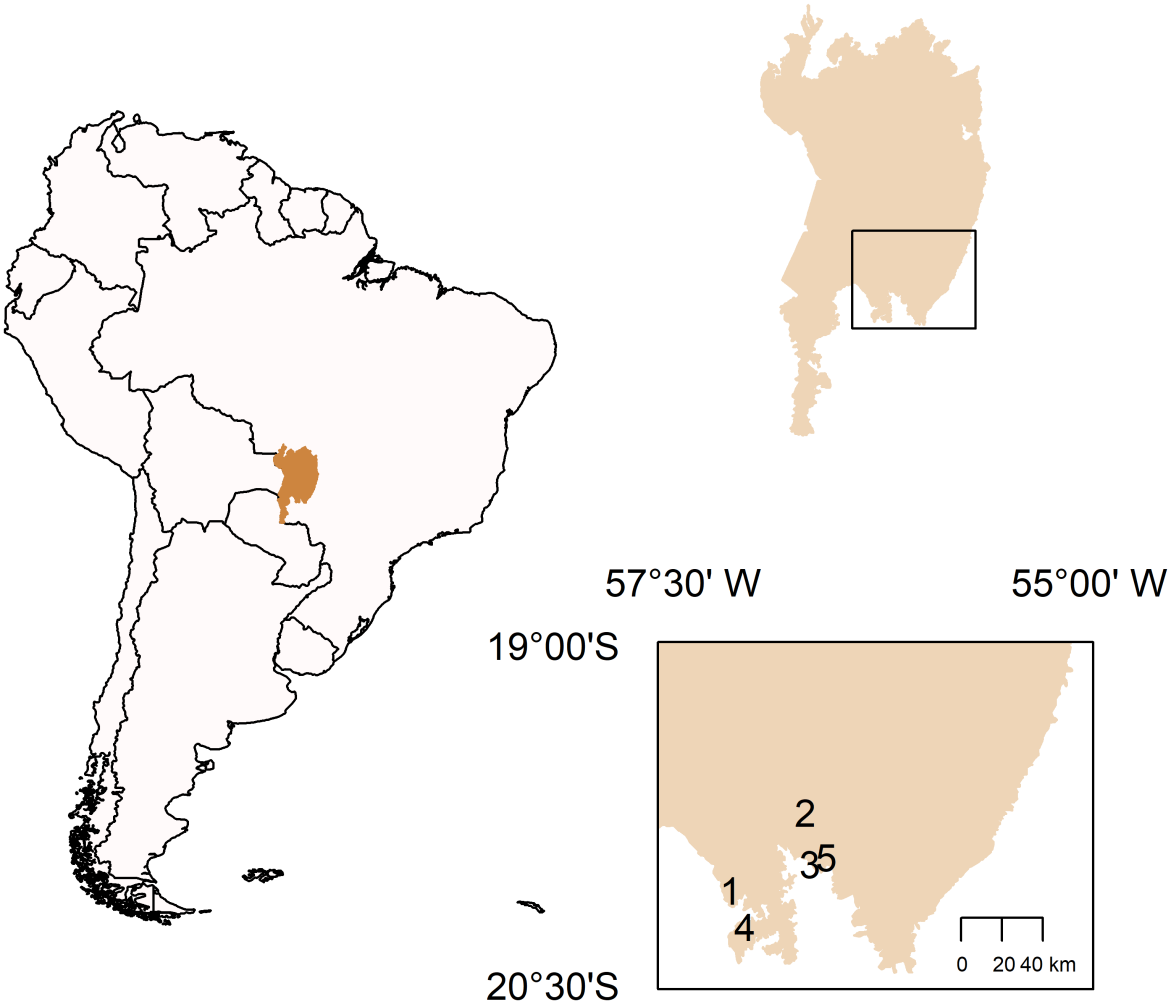
Location of the monitored roosts. Left: Map of South America [34] showing the position of the Pantanal in western Brazil (in light brown) and in the center of the continent. Upper right: The Pantanal of Brazil showing the position of the inset map. Lower right: in the inset, a schematic map showing the locations of the five studied roosts of Blue-fronted Amazons (1–5) in the southern portion of the Pantanal region. Roost 1 (20°05'07” S, 56°36'46” W), Roost 2 (19°44'13” S, 56°19'53” W), Roost 3 (19°57'46” S, 56°18'19” W), Roost 4 (20°14'28” S, 56°36'43” W), and Roost 5 (19°56'04” S, 56°16'45” W).

The distance between the monitored roosts varied from 4.4 km to 63.2 km (X = 36.8 km, SD = 18.2 km) (Fig 1). Three out of the five roosts were located in remnant fragments of semideciduous forest and/or cerrado woodland (Roost 1, Roost 4 and Roost 5). Roost 1 was in the last forest patch within a matrix of approximately 40 km^2^ of irrigated rice, but there were still pristine habitats around this area. Roosts 4 and Roost 5 were within pastures for cattle ranching, but many other patches of woody vegetation remain in their vicinity. Roost 2 was in a continuous and well-preserved riverine forest in the proximity of flooded fields. Roost 3 was in a patch composed of dozens of mango trees (*Mangifera indica*). This roost was contiguous to the worker houses on a farm and was therefore in a very disturbed area. At least two of the roosts (1 and 3) were known to have persisted for decades.

Sporadically we registered the presence of few individuals of Orange-winged Parrots (*Amazona amazonica*) sharing the same perches of the Blue-fronted Amazon in these five roosts. In Roost 3, the presence of a couple of hundreds of Peach-fronted Parakeets (*Eupsittula aurea*) was normal, and they occupied the peripheral area of the roosts. In both cases, we never witnessed any interference or agonist behavior between the species.

### Roost counts

We conducted one count per month in each roost, totaling 249 counts in the five communal roosts within 498 h of observation. Roosts 1–3 were monitored for 61 months from July 2004; Roost 4 was monitored for 38 months from July 2004; and Roost 5 was monitored for 28 months from September 2004. The counts were conducted around sunset, when the parrots arrived at the roosts to spend the night, following the protocol described in previous studies [31, 35]. Two observers, who were positioned at locations (on land or from a boat) 200–700 m apart, counted the different previously defined sectors (e.g. North and South, or East and West), visually delimited by landmarks (e.g. a path of trees, a river channel, etc.). We preferred to perform the counts just during dusk because the parrots leave the roosts rapidly at dawn in flocks that are too large to allow for accurate counting.

The observers simultaneously recorded the number and the size of the flocks arriving at each the roost, in singles, pairs (a unit of two paired parrots), family flocks (three to six individuals flying together), or large flocks that could include many dozens of parrots. The family flocks could usually be distinguished, as the birds flew in close proximity, up to five meters from one another, and the young often interact (e.g., changing their position during flight and playing) and follow the adults. Because these family flocks are composed of the parental pair plus their one to four fledglings (e.g., [17, 36]), we discounted the parental pair in each counted family flock [18] when analyzing the data related to the number of fledglings. The counts were conducted when the sky was clear enough to distinguish the birds in flight, and we avoided adverse climatic conditions, such as rains and strong winds. Data for these counts are available from the protocols.io repository entitled: “Blue-fronted Amazon roosts in five roosts in Brazil Seixas and Mourao 2018” dx.doi.org/10.17504/protocols.io.srped5n available from https://www.protocols.io/view/blue-fronted-amazon-roosts-in-five-roosts-in-brazi-srped5n.

### Data analysis

For every roost, we tabulated the median of the roosting parrots by month and stratified by flock-size, to examine whether the roosts counts varied much throughout the year. Additionally, we used the function *aovp()* in the package lmPerm [37], to determine if the monthly counts of Blue-fronted Amazons on the five roosts pooled together had a seasonal pattern (i.e. the counts varied between the months), and we plotted the counts, which were stratified by flock size, of the five roosts pooled together as a function of month. To evaluate whether every roost site followed the same annual pattern, we applied another permutation ANOVA (aovp), modeling the counts as a function of roosts, months, and the interaction term. Once this analysis indicated that the roosts followed different seasonal patterns, for every roost and every flock-size strata, we applied the “Seasonal and Trend decomposition using Loess” (STL) procedure [38], to decompose the series of counts into three components: trend, seasonal and residual (remaining) variation. We used the *stl()* function in the R computing environment, using the argument *s*.*window = "periodic"* to perform this analysis [39]. The process involve the following steps: (1) the seasonal component for the subseries is obtained by averaging the subseries (i.e. obtaining the mean of all January values, all February, etc.); (2) the original data minus the seasonal component is smoothed by an algorithm of local regression (locally weighted scatterplot smoothing, or loess [40]) to determine the trend; and (3) the remainder component is computed as the residuals from the seasonal plus trend fit. This process is iterated a few times. We applied the STL to examine the counts of the Blue-fronted Amazons landing at each roost stratified by the numbers of birds of the arriving flocks as singletons, pairs, and fledglings in small family flocks, and all of the group sizes of the parrots pooled together. To allow for an easy comparison between the roosts, we used radial plots of the seasonal component resulting from the STL-decomposition analysis, standardized by its range, to summarize the seasonal component of each roost and each parrot flock size in a single figure. Since they were range-standardized, the data presented in these figures are between zero and one. Based on the information provided elsewhere [41], we established a year-round calendar of the breeding cycle of the Blue-fronted Amazon and plotted it together with the radial plots as a guide to facilitate the interpretation of the plots.

As counts and percentages are seldom normally distributed (e.g., [42, 43]), we used the median and interquartile range (IQR) to describe central tendencies and variability along with text and tables. To test for differences in the percentages of the flock-size strata among the roosts, we used one-way permutation ANOVAs [37, 44].

We used the linear models with permutation tests to access (i) the population trends in the five roosts pooled together over the study period, and (ii) the recruitment trends, i.e., the trends of fledglings in the five roosts pooled together over the study period. Finally, we performed a permutation ANCOVA to test whether the number of family flocks varied among the roosts throughout the period of study and used ANCOVA to test whether the mean number of the fledglings in family flocks varied among the roosts and throughout the period of the study. We excluded the data from Roost 5 for these last two analyses because the numbers of parrots in this roost were already known to have decreased during this study time. To check for the assumption of the parallelism of the slopes, we first ran each model, including the interaction term. For each analysis, if this term proven to be nonsignificant, we excluded it and proceeded to run the ANCOVAs.

## Ethics statement

This study was carried out in three private properties: Refúgio Ecológico Caiman, San Francisco Agroecoturismo and Refúgio da Ilha Ecolodge, and their owners explicitly gave permission to one of us (GHFS) to conduct this study on these sites. The study was purely observational and the observers were located at hundreds meters from the birds’ roosts and did not produce noise or act in any way that could alarm the birds. The study did not implied in capture or handling of birds or any kind of habitat manipulation and therefore did not demanded the exam by an Institutional Animal Care and Use Committee.

## Results

### Counts of Blue-fronted Amazons in roosts

**Fig 2:**
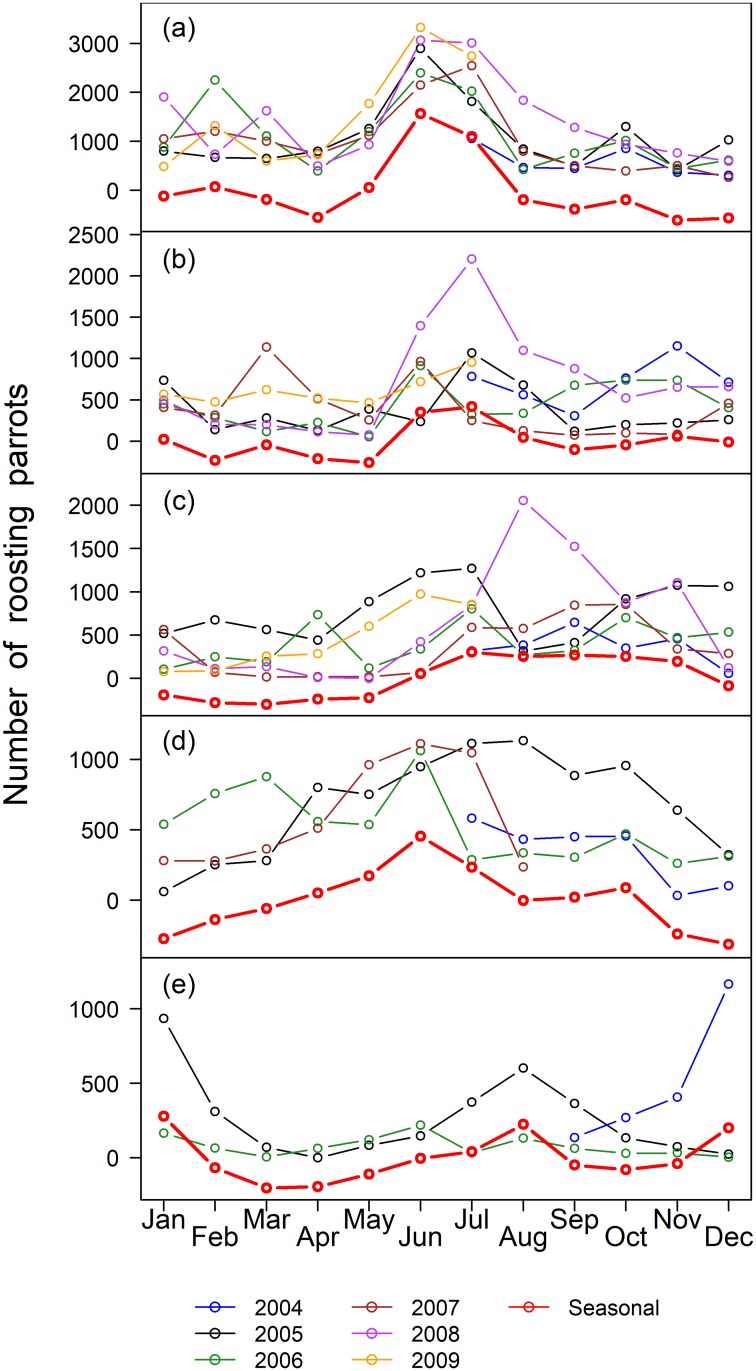
Number of parrots in five roosts in the southern Pantanal of Brazil. Years are coded by colors (see the legend below the plots), and the red line indicate the seasonal component of the counts obtained by STL-decomposition analysis (see Methods). (a) Roost 1, (b) Roost 2, (c) Roost 3, (d) Roost 4, and (e) Roost 5.

**Fig 3:**
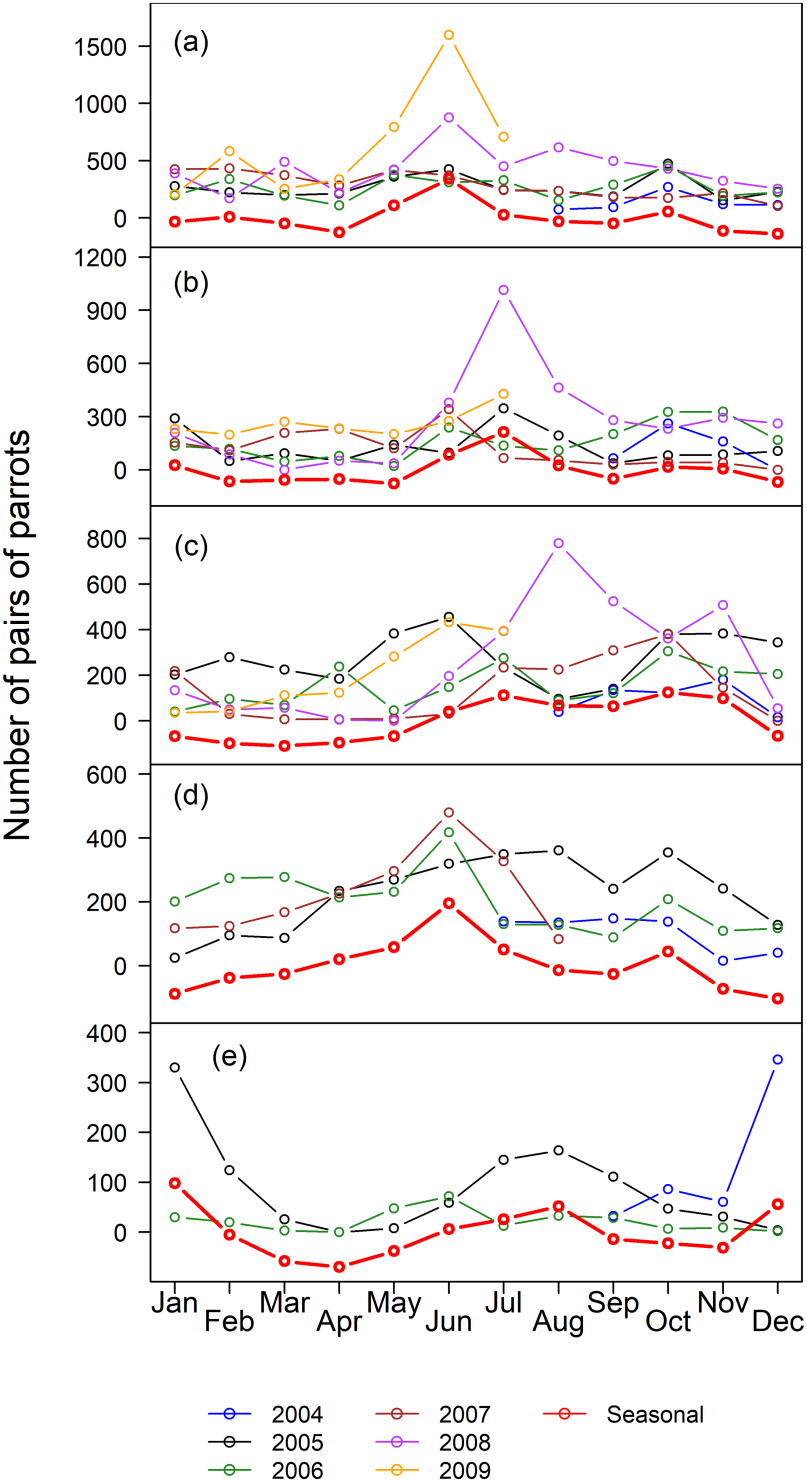
Number of pairs of parrots in five roosts in the southern Pantanal of Brazil. Years are coded by colors (see the legend below the plots), and the red line indicate the seasonal component of the counts obtained by STL-decomposition analysis (see Methods). (a) Roost 1, (b) Roost 2, (c) Roost 3, (d) Roost 4, and (e) Roost 5.

**Fig 4:**
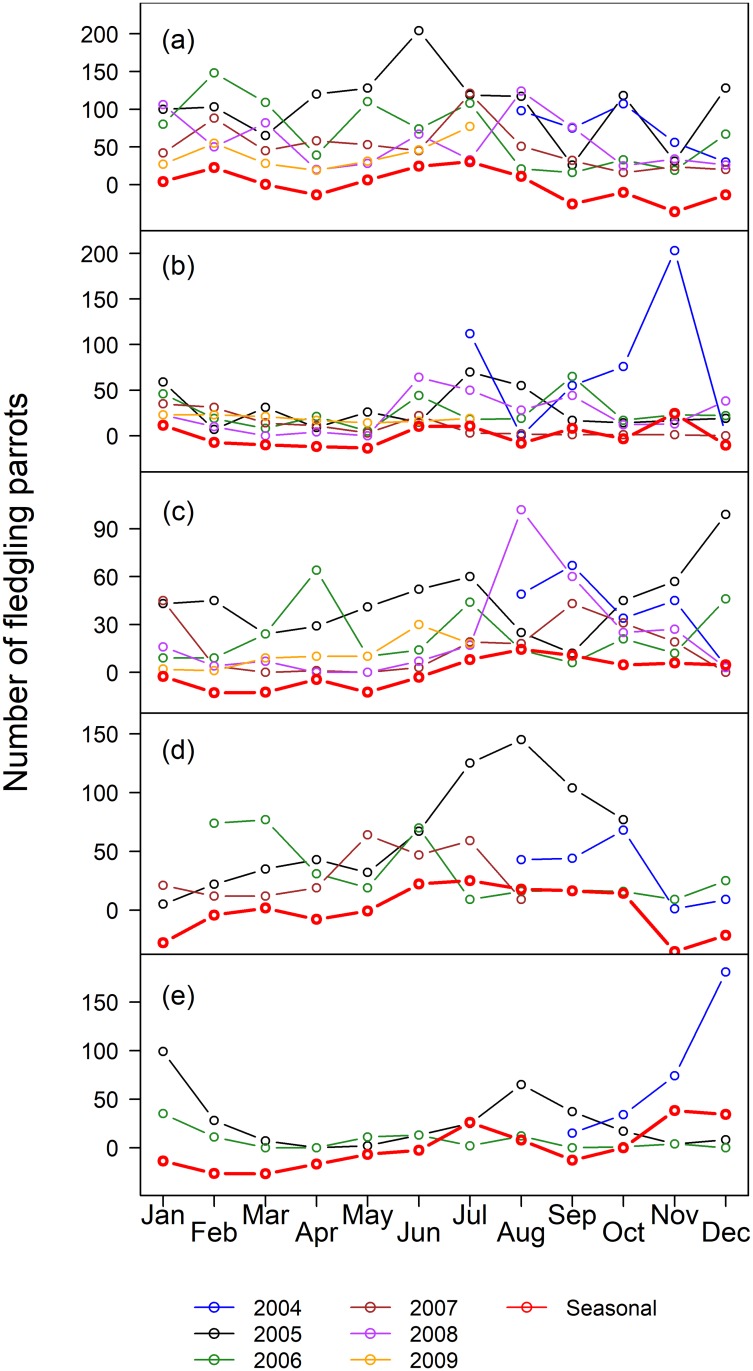
Number of fledgling parrots in five roosts in the southern Pantanal of Brazil. Years are coded by colors (see the legend below the plots), and the red line indicate the seasonal component of the counts obtained by STL-decomposition analysis (see Methods). (a) Roost 1, (b) Roost 2, (c) Roost 3, (d) Roost 4, and (e) Roost 5.

**Fig 5:**
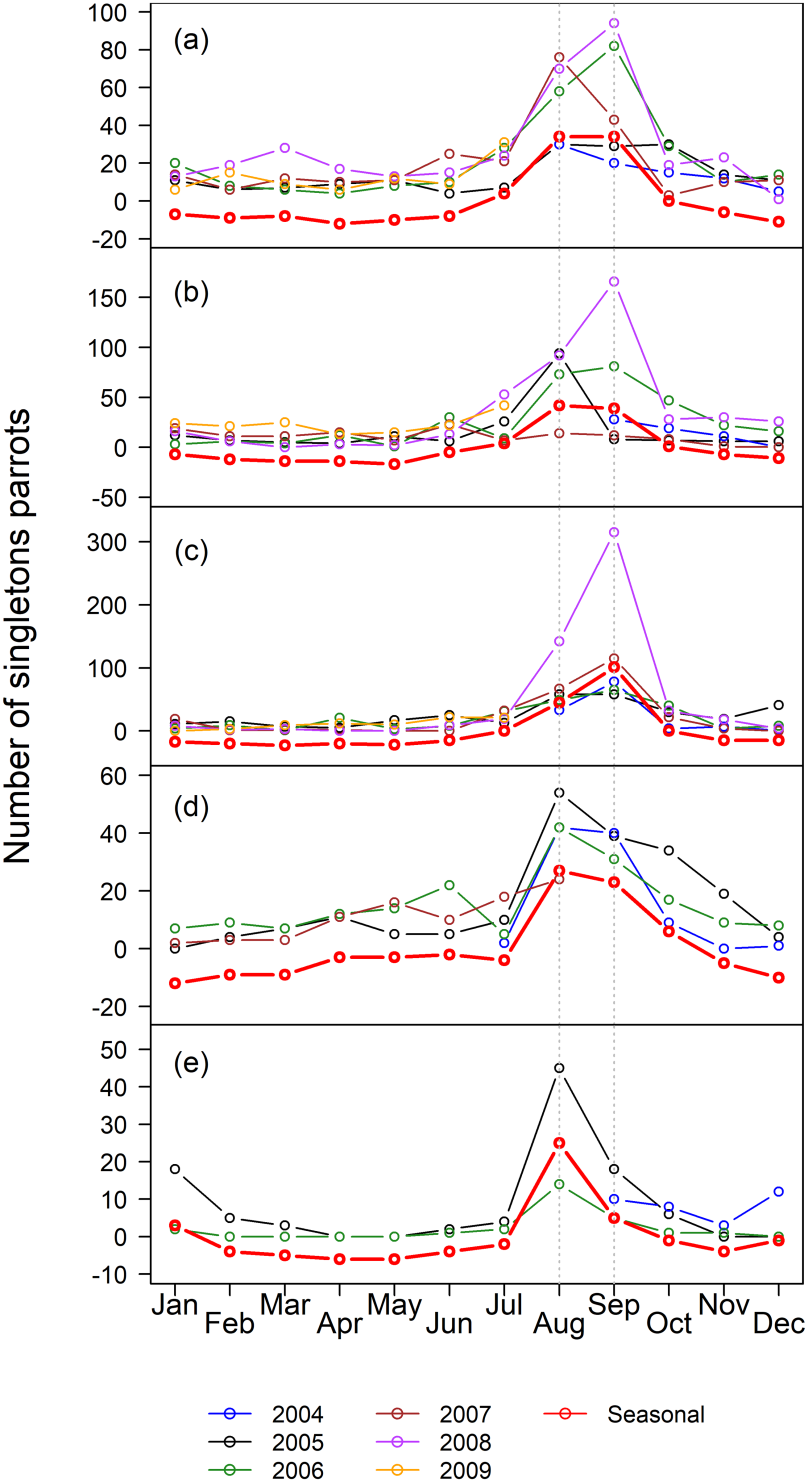
Number of singletons parrots in five roosts in the southern Pantanal of Brazil. Years are coded by colors (see the legend below the plots), and the red line indicate the seasonal component of the counts obtained by STL-decomposition analysis (see Methods). (a) Roost 1, (b) Roost 2, (c) Roost 3, (d) Roost 4, and (e) Roost 5. Vertical dotted lines indicates the months of August and September.

**Fig 6:**
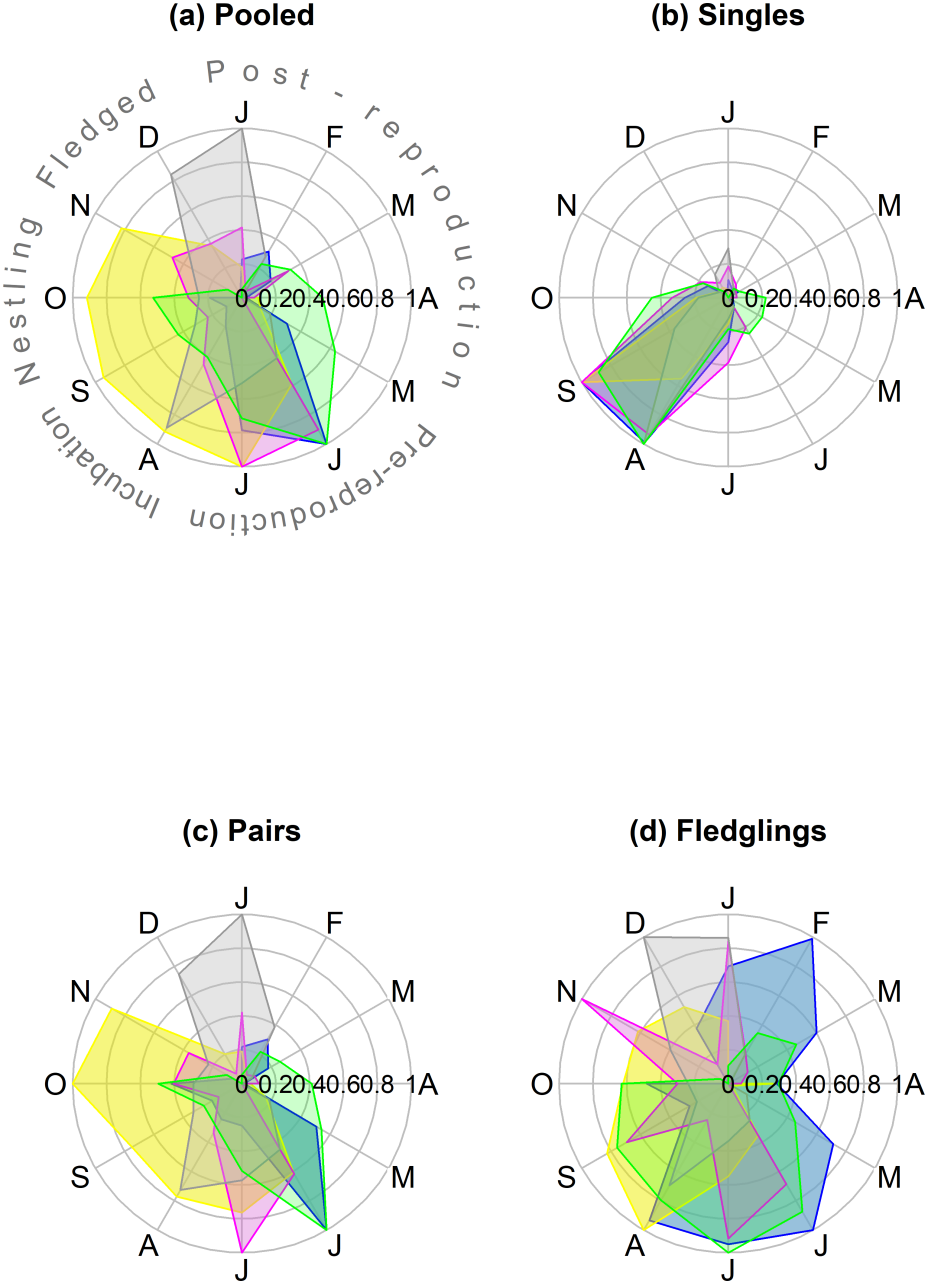
Radial plots of the seasonal component resulting from the decomposition analysis of monthly counts of Blue-fronted Amazons (see Methods) stratified by group size in five roosts in the southern Pantanal of Brazil. Seasonal components were range standardized in order to obtain values of between one and zero. “All Parrots” refers to the numbers of parrots arriving at each roost irrespective of the flock size, “Pairs” refers to the number of pairs, “Singles” refers to the number of singletons, and “Fledglings” refers to the number of parrots arriving at the roosts in family groups of three to six birds. Blue = Roost 1, violet = Roost 2, yellow = Roost 3, green = Roost 4, gray = Roost 5. Information about the reproductive cycle of the Blue-parrot Amazon [41] appears in the upper left graph. Counts were carried out from July 2004 to July 2009. Graphical results of the STL decomposition analyses of each flock-size strata in each roost are supplied (S2–S21 Figs).

The pooled counts of Blue-fronted Amazons in the five roosts for each month in the southern Pantanal ranged from 622 (Avril 2008) to 6064 (July 2008) (S1 Table), with a median of 2302 (IQR = 1526) parrots. The median number of parrots by month varied substantially between roosts and among the roosts, ranging from 24 to 2896 parrots (Table 1). The time of the year of the minimum counts, based on the median, varied among roosts, and the maximum counts occurred in June for three of the five roosts (Table 1), suggesting a seasonal component to the counts. In general, the pooled counts in the five roosts varied with the months of the counts (df = 11, 49, p_permuted <_ 0.001, r^2^ = 0.62), and were higher in Jun-Jul for all roosting parrots, pairs and large flocks (S1 Fig), but were higher in Aug-Sep for singletons. The fledglings did not showed an obvious pattern. However, the seasonal patterns of roost attendance varied significantly among roosts (df = 44, 189, p_permuted_ < 0.001, R^2^ = 0.67), indicating that the time series of each roost must be examined separately. In fact, different roosts had different dynamics, in relation to the all-parrots (Fig 2), the pairs of parrots (Fig 3) and the fledglings (Fig 4), but not for the singletons (Fig 5). The singletons had a strong seasonal pattern, with maximum numbers occurring in Aug-Sep for every roost (Fig 6).

**Table 1:** Median of monthly counts in five roosts of Blue-fronted Amazon in the Pantanal of Brazil. Maximum medians are indicated by shaded cells, and minimum medians are underlined. Roosts 1–3 were monitored for 61 months starting in July 2004. Roost 4 was monitored for 38 months starting in July 2004, and Roost 5 was monitored for 28 months starting in September 2004.

Roost	Jan	Feb	Mar	Apr	Mai	Jun	Jul	Aug	Sep	Oct	Nov	Dec
1	885	1207	1001	729	1201	2896	2287	796	505	940	447	593
2	497	285	282	228	258	915	869	562	309	524	653	460
3	318	112	191	286	119	422	826	385	645	855	470	287
4	281	279	365	559	752	1063	815	385	451	471	261	313
5	310	188	38	32	103	147	110	168	100	202	200	24
Pooled_1-5_	2726	2156	1860	1979	2232	5288	4640	1950	2011	3065	2155	1722

The STL-generated seasonal components of the counts, which appeared in red in Figs 2–5, are easier to interpret in relation to the breeding cycle of the parrots in range-standardized circular plots (Fig 6). Roosts 1 and 2 reached the maximum values for all parrots in Jun-Jul, which coincided with the pre-reproduction period, and the numbers fell abruptly in Aug-Oct during the incubation, hatching and nestling growth periods (Fig 6a). The minimum counts occurred in April. The parrots consistently used Roost 3 from Jun-Nov, which includes part of the pre-reproduction period and most of the reproductive period. As in Roost 1, Roost 4 also showed maximum numbers in June, but in contrast to the former, an increasing number of parrots used Roost 4 from January to June, including a large part of the post-breeding period and part of the pre-reproduction period. The minimum use of this roost occurred during Dec-Jan when it was expected that most of the fledglings had already left the nests. It is difficult to interpret the figures of Roost 5 since it tended to disappear throughout the study period. Loosely, we observed two modes: one from December 2004 to January 2005 (post-fledged) and another in August 2005, at the beginning of the incubation period. Therefore, the Blue-fronted Amazon used different roosts during different parts of its breeding cycle.

### Counts of Blue-fronted Amazons in roosts stratified by flock size

The percentage of median counts of singletons during the incubation period (August-September) in relation to the median of the counts of the total numbers of parrots roosting in June-July varied from 1.3% for Roost 1 in 2005 to 35.9% for Roost 3 in 2008 (Table 2). The percentages of singletons differed among the roost sites (df = 4, 16; p_permutated_ = 0.012, r^2^ = 0.53), with the lowest median percentage occurring in Roost 1 and the highest median percentage occurring in Roost 3. There was a strong seasonal pattern in the single-parrot counts, with the maximum numbers occurring during August-September in every roost (Fig 6b), corresponding to the incubation period. The minimum numbers occurred in February-March, matching with the post-reproductive period.

**Table 2:** Counts of Blue-fronted Amazons in five roosts monitored in the southern Pantanal of Brazil. The percentages of these counts stratified by group size are also shown. “All parrots” refers to the average counts from June-July. “Singles” refers to the average percentages of singleton parrots from August-September in relation to “All parrots”. “Paired” and “Fledglings” refer to the percentages of the mean counts from June-July of individuals belonging to these strata in relation to “All parrots”. Counts were carried out from July 2004 to July 2009.

Roost	Stratum	2004	2005	2006	2007	2008	2009	Median	IQR
1	All parrots	1059	2356	2213	2348	3038	3032	2352	617
	Singles (%)	2.4	1.3	3.2	2.6	2.7	-	2.6	0.3
	Paired (%)	-	28.4	28.8	26.2	43.7	76.2	28.8	15.3
	Fledglings (%)	1.5	6.9	4.1	3.5	1.6	2	2.8	2.2
2	All parrots	784	652	620	608	1802	836	718	195
	Singles (%)	1.8	7.8	12.4	2.1	7.2	-	7.2	5.7
	Paired (%)	-	67.9	60.5	67.1	77.3	84.1	67.9	10.2
	Fledglings (%)	-	6.5	5	2.1	3.2	2.1	3.2	2.9
3	All parrots	318	1246	571	328	635	913	603	455
	Singles (%)	17.6	4.7	10	27.7	35.9	-	17.6	17.7
	Paired (%)	-	55.3	74.1	80.5	92.8	90.5	80.5	16.4
	Fledglings (%)	3	4.5	5.1	3.4	1.9	2.6	3.2	1.5
4	All parrots	583	1030	676	1078	-	-	853	389
	Singles (%)	7	4.5	5.3	2.2	-	-	4.9	1.8
	Paired (%)	47.7	64.9	81.1	74.9	-	-	69.9	15.8
	Fledglings (%)	-	9.3	5.8	4.9	-	-	5.8	2.2
5	All parrots	-	260	127	-	-	-	194	66
	Singles (%)	-	12.3	7.9	-	-	-	10.1	2.2
	Paired (%)	-	78.3	66.9	-	-	-	72.7	5.8
	Fledglings (%)	-	7.3	5.9	-	-	-	6.6	0.7
Pooled_1-5_	All parrots	2744	5544	4207	4362	5475	4781	4571	1056
	Singles (%)	5.0	3.9	5.9	4.3	8.0	-	5	1.6
	Paired (%)	-	48.2	49.2	48.0	60.4	80.3	49.2	12.2
	Fledglings (%)	-	6.8	6.5	3.6	2.2	2.1	3.6	4.3

In general, parrots in pairs dominated the flock-size composition in roosts on an annual basis (Table 2), ranging from 26.2% of the individuals (Roost 1, year 2007) to 92.8% (Roost 3, year 2008). Roosts differed in terms of the proportion of paired parrots (df = 4, 16, p_permutated_ = 0.015, r^2^ = 0.67), with Roost 1 generally having the lowest medians. The seasonal pattern of pair counts (Figs 3c and 6c) tended to follow the pattern of all Blue-fronted Amazons in roosts (Fig 3a).

The annual median percentage of counts of fledglings varied from 1.5% in Roost 1 (2004) to 9.3% in Roost 4 (2005) (Table 2). There was a nonsignificant tendency of the percentage of fledglings to vary among roosts (df = 4, 17, p_permuted_ = 0.054, r^2^ = 0.42), with lower percentages occurring in Roost 1. The seasonal patterns were complex and varied among the roosts (Figs 3d and 6d).

It is not easy to detect the movement of individuals between roosts from the counts. However, the changes in the modes of pairs of parrots from Roost 2 in July 2008 (Fig 3b) to Roost 3 in August 2008 (Fig 3c) suggest that hundreds of pairs of parrots moved from Roost 2 to Roost 3 at that time. Similarly, the increase in the mode of pairs observed in the next year (June 2009) in Roost 1 (Fig 3a) suggests that this roost received hundreds of pairs of parrots which possibly originated from Roosts 2 and 3.

### Trends

**Fig 7:**
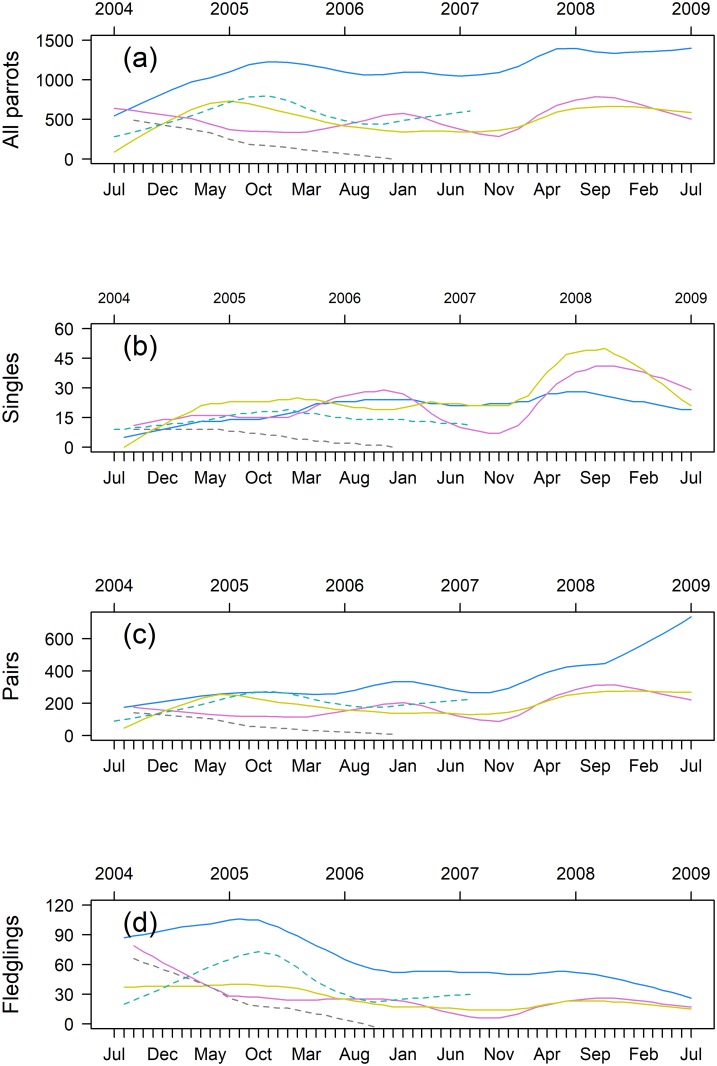
Trends obtained by the decomposition analysis of the monthly counts of Blue-fronted Amazons in five roosts (Roosts 1–5) in the southern Pantanal of Brazil, stratified by the flock sizes of parrots. (a) Counts of all parrots pooled together. (b) Counts of singleton parrots. (c) Counts of pairs of parrots. (d) Counts of family groups (3–6 birds). Blue = Roost 1, violet = Roost 2, yellow = Roost 3, green = Roost 4, and gray = Roost 5. Counts were carried out from July 2004 to July 2009.

The long-term trends of the parrots (Fig 7a) suggest that all the roosts roughly followed similar patterns, except for Roost 5, which suffered a marked decline in numbers throughout the years and tended to disappear. In December 2006, only four parrots were using Roost 5, and in May 2007, no parrots were using that roost. The numbers of parrots in the other roosts tended to fluctuate around their own averages for most of the time between 2005 and 2007, but the three roosts that we monitored after August 2007 (Roosts 1–3) experienced marked increases in number during 2008. After this increase in the number of birds, Roosts 2 and 3 tended to return to their average numbers, but Roost 1 seems to have reached a higher baseline.

The trends of singletons (Fig 7b) in Roost 2 and Roost 3 responded to the 2008 input and tended to return to their baseline, but the trends of singletons at Roost 1 were relatively insensitive to the 2008 input. The trends of pairs in Roost 1 markedly differed from the trends of pairs at other roosts, especially because the increase began around November 2007 and persisted to the end of the study in mid-2009 (Fig 7c). The trends of pairs at roosts 2–4 tended toward stability. The trends of the fledglings visually differed from those of all parrots for most roosts, with an overall tendency for a decrease prevailing from the end of 2005 through the study period, except for a small recovery during 2008 (Fig 7d). The counts of parrots in the five roosts pooled together did not change throughout the study (F_(1, 59)_ = 0.097, p = 0.76), but the counts of fledglings changed over time (F_(1,56)_ = 63.51, p < 0.001, r^2^ = 0.53), with a slope β = -4.127, i.e. decreasing by approximately 50 fledglings per year in the studied population. This accounted a decrease in both the numbers of family flocks and the size of these flocks. The number of family flocks in Roosts 1 to 4 decreased throughout the study period (F_(4, 214)_ = 20.29, = <0.001) with a slope β = -0.29 (S22 Fig). The overall mean number of fledglings in family flocks was 1.51 birds (SD = 0.35). The mean number of fledglings in the family flocks varied with roosts (F_(3, 203)_ = 2.774; p = 0.043) and decreased throughout the study period (F_(1, 203)_ = 60.819, p < 0.001, R^2^ = 0.25), with a slope β = -0.008 (t = -7.775, df = 203, p < 0.001) (S23 Fig).

## Discussion

Many studies have used the counts of roosting parrots to make inferences about their population numbers and reproductive dynamics [25, 27, 45, 46]. Some of these studies have covered a relatively short period and/or have been limited to a single roost (e.g., [28, 47]) or relatively small, space-restricted total populations [48, 49]. We surveyed five roosts of Blue-fronted Amazons located in an area of approximately 2700 km^2^ in the southern Pantanal of Brazil for 28–61 months. June was the month with a higher number of roosting parrots with a median of 5288 birds, but up to 6064 parrots used the roosts in July 2008, when only three of the five studied roosts were counted. However, this population is not closed in any sense, as large areas around the roosts are suitable habitat for the Blue-fronted Amazon, and we had some evidence that the parrots can move from one roost to another.

The breeding period is often related to a decrease in the number of roosting parrots (e.g., [27, 28, 47, 50]), and it is thought to be the primary factor that determines the fluctuation in the number parrots in roosts [15]. Some authors [27] observed that one member of an incubating pair of Red-lored Amazons (*Amazona autumnalis*), presumably the male, usually returned to the communal roost and stated that the numbers of roosting singletons should increase during this period. This is also known to occur with the Blue-fronted Amazon. Singletons occurred in high numbers in August and September at all monitored roosts and were almost absent during the rest of the year. Consequently, the median proportion of the singletons roosting in August-September in relation to the number of parrots roosting in June-July would be a reasonable estimate of the minimum proportion of pairs of parrots attempting to reproduce each year in relation to the total numbers of roosting parrots. This approach can be useful for other roosting birds, if one bird of the incubating pair is known to return every night to the communal roost, while the another parental remains in their cavity or nest. In addition, one of us previously estimated a median of 0.9 young Blue-fronted Amazons fledged per laying female per year in that area in a series of twelve years of data [51]. This estimate is very close to the 0.95 fledglings per laying female per year found for the same species in the Chaco of Argentina [52]. Therefore, the proportion of singletons would also indicate the proportion of fledged young each year. In our study, the percentages of singletons varied among the roosts, suggesting some specialization among roosts, and ranged from a median of approximately 2.6% for Roost 1 to a median of approximately 17.6% for the more reproductively specialized roost (Roost 3). Pooling the roosts together, we would expect a median increase of approximately 206 fledglings (4.5%) in a given year. These estimates are at the same magnitude as those found in other studies that focus on nonprotected populations of parrots: 2.3–4.6% for Red-tailed Parrots (*Amazona brasiliensis*) [28], 12.5% for Yellow-naped Parrots (*Amazona auropalliata*) [18] and 14% for Red-fronted Macaws (*Ara rubrogenys*) [53]. However, most of the authors (e.g., [28, 29]) used the counts of young in the family groups to estimate recruitment a few months after the fledging period. Using this approach, we would expect recruitment rates of 2.8% to 6.6% per year or a median of approximately 165 six-month-old young roosting in the five studied roosts pooled together every year. The difference of 20–25% between the estimates of recruitment based on the proportion of singletons and based on the proportion of fledglings could be accounted for by the mortality of the fledged young over time between the period of fledging (Nov-Dec) and period of the maximum number of birds in roosts (Jun-Jul). Some authors [18] recognized that such post-fledging mortality in parrots might be considerable (but see also [54]), and it could reach up to 35% for Great Green Macaws (*Ara ambigua*) during the first year after fledging. Therefore, the use of the singleton counts to access the fledging rate and family flock counts to access the post-fledging recruitment can provide insights into post-fledging mortality rates. However, some caution is needed, because if young disperse into nonmonitored roosts, or if two or more family flocks often join to form a large flock, small flocks would not always consist of a breeding pair plus its young from that year [18]. Hence, such post-fledging mortality rate estimates should be considered in the light of the knowledge of the biology of the target species.

The predominance of pairs in the communal roosts seems to be the rule among parrots and has been reported for other Psittacidae species (e.g., [15, 55]). Pairs are thought to be the fundamental unit of the social structure of parrots [55, 56], and in fact, paired Blue-fronted Amazons generally dominate the flock-size structure of the studied roosts. However, the proportion of paired parrots varied among roosts; it was lower in Roost 1 and higher in Roost 3, reinforcing the conclusion of the existence of different types of roosts. In our case, except for the concordance of the presence of singletons in August-September, the monitored Blue-fronted Amazon roosts showed different dynamics throughout the year. Other studies [25] also found different types of roosts in the Red-spectacled Amazon and classified them as pre-reproductive roosts (Jul-Sep), roosts associated with the reproduction period (Oct-Dec), roosts associated with the post-reproduction period (Jan-Feb), and roosts associated with the fruiting period of Paraná pine (*Araucaria angustifolia*) (Mar-Jun). The latter roost type is hundreds of kilometers from the former types, and in these locations, many patches with a high concentration of Paraná pine trees are available. For Blue-fronted Amazons from the Pantanal, although reproductive singletons occurred in every studied roost, we could also identify some roosts that were associated with the pre-reproductive period (Roost 1, Roost 2, and Roost 4), and one roost that was more associated with the reproductive period (Roost 3). This is consistent with the moves of hundreds of pairs of parrots from the pre-reproductive Roost 2 to the reproductive Roost 3 observed in 2008. In addition, the numbers of singletons, which indicate the number of incubating couples, were higher in Roost 3 than in the other roosts, during that year. The Roost 5 had the higher numbers of pairs and fledglings in December and January, which was what we would expect for a post-reproductive roost. However, this roost tended to disappear throughout the study period, preventing us from classifying it in relation to the breeding cycle. Unlike in southern Brazil, where there is a concentrated and explosive increase in food availability with the fruiting of the Paraná pine, in the Pantanal, the food resources are widely dispersed during that time of the year (Feb-May) [49, 57, 58], which coincides with the lower number of parrots using the monitored roosts. At this time of year, it could be energetically favorable for the parrots to avoid long displacements to the large roosts and find alternative shelters near the foraging areas to spend the nights.

### Trends and implications for management

The trends in the parrot counts suggest that at least three of the five roosts received an input of birds during 2008, which cannot be explained by the decrease in the number of parrots in Roost 5, which occurred at least a year before. This result is consistent with an open population, and the monitored roosts seem to exchange individuals among themselves and with nonmonitored roosts. The input of singletons was less pronounced in the pre-reproductive Roost 1, and pairs were more pronounced there than in the other monitored roosts, which indicated that this exchange of individuals among roosts was stratified by age and/or reproductive condition, as with other parrots [25]. In general, the numbers of pairs of parrots and singletons using the roosts increased or fluctuated around a baseline, indicating that at least approximately the same number of pairs were attempting to reproduce each year. Despite this, the trend of fledglings was declining fast over time. Both, the number of family flocks and number of fledglings in these flocks decreased over the monitoring period. Such a scenario, of the stability of the reproductive fraction of the population allied with the decreasing recruitment of young, may occur in long-living organisms such as parrots. This could be especially worrying, as it can biased the perception of the management authorities in relation to threatened populations. Different factors, such as poaching, climatic events, diseases, or habitat destruction, which are eventually associated with other processes, could jeopardize the recruitment. Although a recent study have indicated that the capture for the local pet trade was the main factor threatening most of the studied Neotropical parrots populations [2], we have no direct evidence that poaching has been a severe threat in that area. In addition, we did not note an increase in the frequency of severe climatic events during the monitoring time, and we have not perceived signs of serious diseases among these parrots. On the other hand, we do observe the cutting of woody vegetation patches usually followed by burnings, which leads to the direct destruction of parrot’s cavities and broods within these cavities, and a reduction in food availability for parrots as well as for their predators. Thus, the forest clearing can also lead to an increase in the predation rates of the remaining parrots’ broods, as the predators intensify their search for food.

In addition to actions that minimize habitat destruction in reproduction areas, obviously, the protection of large and longstanding communal roosts is an important measure to protect the wild populations of Blue-fronted Amazons inhabiting the Pantanal. Every monitored roosts has at least some birds in each age or reproductive condition strata throughout the year: therefore, each one of these roosts covers all breeding cycles. However, the fact that the roosts are different, and some are more prone to be used in a particular part of the life cycle of the parrots, suggests that it will be more effective for the long term conservation of the Blue-fronted Amazon to protect a carefully chosen set of complementary roosts, in light of the biology of parrots.

## Supporting information

S1 Fig Pooled counts of Blue-fronted Amazons in five roosts in the Pantanal of Brazil. (a) “All Parrots” refers to the numbers of parrots arriving the roosts irrespective of the flock size, (b) “Singletons” refers to the number of singletons, (c) “Pairs” refers to the number of pairs, (d) “Fledglings” refers to the number of fledgling parrots in family groups of three to six birds, and (e) “Large flocks” refers to parrots in flocks larger than six parrots. Counts were carried out from July 2004 to July 2009.
(PDF)

S2 Fig Decomposition analysis of the monthly counts of Blue-fronted Amazons in Roost 1 in the southern Pantanal of Brazil. (a) Monthly counts of parrots. (b) Seasonal pattern. (c) Seasonally adjusted trend of parrots along the study time. (d) The remainder. Counts were carried out from July 2004 to July 2009.
(PDF)

S3 Fig Decomposition analysis of the monthly counts of the singletons of Blue-fronted Amazons in Roost 1 in the southern Pantanal of Brazil. (a) Monthly counts of parrots. (b) Seasonal pattern. (c) Seasonally adjusted trend of parrots along the study time. (d) The remainder. Counts were carried out from July 2004 to July 2009.
(PDF)

S4 Fig Decomposition analysis of the monthly counts of the pairs Blue-fronted Amazons in Roost 1 in the southern Pantanal of Brazil. (a) Monthly counts of pairs parrots. (b) Seasonal pattern. (c) Seasonally adjusted trend of pairs of parrots along the study time. (d) The remainder. Counts were carried out from July 2004 to July 2009.
(PDF)

S5 Fig Decomposition analysis of the monthly counts of Blue-fronted Amazons fledglings in Roost 1 in the southern Pantanal of Brazil. (a) Monthly counts of parrots. (b) Seasonal pattern. (c) Seasonally adjusted trend of parrots along the study time. (d) The remainder. Counts were carried out from July 2004 to July 2009.
(PDF)

S6 Fig Decomposition analysis of the monthly counts of Blue-fronted Amazons in Roost 2 in the southern Pantanal of Brazil. (a) Monthly counts of parrots. (b) Seasonal pattern. (c) Seasonally adjusted trend of parrots along the study time. (d) The remainder. Counts were carried out from July 2004 to July 2009.
(PDF)

S7 Fig Decomposition analysis of the monthly counts of the singletons of Blue-fronted Amazons in Roost 2 in the southern Pantanal of Brazil. (a) Monthly counts of parrots. (b) Seasonal pattern. (c) Seasonally adjusted trend of parrots along the study time. (d) The remainder. Counts were carried out from July 2004 to July 2009.
(PDF)

S8 Fig Decomposition analysis of the monthly counts of the pairs of Blue-fronted Amazons in Roost 2 in the southern Pantanal of Brazil. (a) Monthly counts of pairs of parrots. (b) Seasonal pattern. (c) Seasonally adjusted trend of parrots along the study time. (d) The remainder. Counts were carried out from July 2004 to July 2009.
(PDF)

S9 Fig Decomposition analysis of the monthly counts of Blue-fronted Amazons fledglings in Roost 2 in the southern Pantanal of Brazil. (a) Monthly counts of parrots. (b) Seasonal pattern. (c) Seasonally adjusted trend of parrots along the study time. (d) The remainder. Counts were carried out from July 2004 to July 2009.
(PDF)

S10 Fig Decomposition analysis of the monthly counts of Blue-fronted Amazons in Roost 3 in the southern Pantanal of Brazil. (a) Monthly counts of parrots. (b) Seasonal pattern. (c) Seasonally adjusted trend of parrots along the study time. (d) The remainder. Counts were carried out from July 2004 to July 2009.
(PDF)

S11 Fig Decomposition analysis of the monthly counts of the singletons of Blue-fronted Amazons in Roost 3 in the southern Pantanal of Brazil. (a) Monthly counts of parrots. (b) Seasonal pattern. (c) Seasonally adjusted trend of parrots along the study time. (d) The remainder. Counts were carried out from July 2004 to July 2009.
(PDF)

S12 Fig Decomposition analysis of the monthly counts of the pairs of Blue-fronted Amazons in Roost 3 in the southern Pantanal of Brazil. (a) Monthly counts of pairs of parrots. (b) Seasonal pattern. (c) Seasonally adjusted trend of parrots along the study time. (d) The remainder. Counts were carried out from July 2004 to July 2009.
(PDF)

S13 Fig Decomposition analysis of the monthly counts of Blue-fronted Amazons fledglings in Roost 3 in the southern Pantanal of Brazil. (a) Monthly counts of parrots. (b) Seasonal pattern. (c) Seasonally adjusted trend of parrots along the study time. (d) The remainder. Counts were carried out from July 2004 to July 2009.
(PDF)

S14 Fig Decomposition analysis of the monthly counts of Blue-fronted Amazons in Roost 4 in the southern Pantanal of Brazil. (a) Monthly counts of parrots. (b) Seasonal pattern. (c) Seasonally adjusted trend of parrots along the study time. (d) The remainder. Counts were carried out from July 2004 to August 2007.
(PDF)

S15 Fig Decomposition analysis of the monthly counts of the singletons of Blue-fronted Amazons in Roost 4 in the southern Pantanal of Brazil. (a) Monthly counts of parrots. (b) Seasonal pattern. (c) Seasonally adjusted trend of parrots along the study time. (d) The remainder. Counts were carried out from July 2004 to August 2007.
(PDF)

S16 Fig Decomposition analysis of the monthly counts of the pairs of Blue-fronted Amazons in Roost 4 in the southern Pantanal of Brazil. (a) Monthly counts of pairs of parrots. (b) Seasonal pattern. (c) Seasonally adjusted trend of parrots along the study time. (d) The remainder. Counts were carried out from July 2004 to August 2007.
(PDF)

S17 Fig Decomposition analysis of the monthly counts of Blue-fronted Amazons fledglings in Roost 4 in the southern Pantanal of Brazil. (a) Monthly counts of parrots. (b) Seasonal pattern. (c) Seasonally adjusted trend of parrots along the study time. (d) The remainder. Counts were carried out from July 2004 to August 2007.
(PDF)

S18 Fig Decomposition analysis of the monthly counts of Blue-fronted Amazons in Roost 5 in the southern Pantanal of Brazil. (a) Monthly counts of parrots. (b) Seasonal pattern. (c) Seasonally adjusted trend of parrots along the study time. (d) The remainder. Counts were carried out from September 2004 to December 2006.
(PDF)

S19 Fig Decomposition analysis of the monthly counts of the singletons of Blue-fronted Amazons in Roost 5 in the southern Pantanal of Brazil. (a) Monthly counts of parrots. (b) Seasonal pattern. (c) Seasonally adjusted trend of parrots along the study time. (d) The remainder. Counts were carried out from September 2004 to December 2006.
(PDF)

S20 Fig Decomposition analysis of the monthly counts of the pairs of Blue-fronted Amazons in Roost 5 in the southern Pantanal of Brazil. (a) Monthly counts of pairs of parrots. (b) Seasonal pattern. (c) Seasonally adjusted trend of parrots along the study time. (d) The remainder. Counts were carried out from September 2004 to December 2006.
(PDF)

S21 Fig Decomposition analysis of the monthly counts of Blue-fronted Amazons fledglings in Roost 5 in the southern Pantanal of Brazil. (a) Monthly counts of parrots. (b) Seasonal pattern. (c) Seasonally adjusted trend of parrots along the study time. (d) The remainder. Counts were carried out from September 2004 to December 2006.
(PDF)

S22 Fig Partial residuals of the permuted ANCOVA model relating the number of family flocks of Blue-fronted Amazons with (a) the monitoring time and (b) roosts. Counts were carried out in four roosts in the Pantanal of Brazil from September 2004 to December 2006. Bands represent the 95% confidence interval.
(PDF)

S23 Fig Partials residuals of the ANCOVA model relating the mean number of fledglings of Blue-fronted Amazons in family groups with (a) the monitoring time and (b) roosts. Counts were carried out in four roosts in the Pantanal of Brazil from September 2004 to December 2006. Bands represent the 95% confidence interval.
(PDF)

S1 Table Pooled counts of Blue-fronted Amazons. Counts were performed in five roosts by month in the southern Pantanal of Brazil from July 2004 to July 2009. “Roost” is the roost id (1–5), “all.parrots” refers to the number of parrots arriving at roosts irrespective of the group size, “singles” refer to the numbers of singletons, “pairs” refer to numbers of dyads arriving the roosts. “Fledglings” are the number of young fledged in family flocks, “family.flocks” refers to the number of parrots arriving the roosts in small flocks of three to six birds, and “large.flocks” refer to number of the number of parrots arriving the roosts in large flocks (i.e. > 7 parrots).
(XLSX)
