# Supplementary material for: Communal roosts of the Blue-fronted Amazons (Amazona aestiva) in a large tropical wetland: Are they of different types?
Source: PLoS One. 2018 Oct 17;13(10):e0204824. doi: 10.1371/journal.pone.0204824 (PMC6192593; doi:10.1371/journal.pone.0204824)
Supplement: S1 Fig — (a) “All Parrots” refers to the numbers of parrots arriving the roosts irrespective of the flock size, (b) “Singletons” refers to the number of singletons, (c) “Pairs” refers to the number of pairs, (d) “Fledglings” refers to the number of fledgling parrots in family groups of three to six birds, and (e) “Large flocks” refers to parrots in flocks larger than six parrots. Counts were carried out from July 2004 to July 2009. (PDF) [file pone.0204824.s001.pdf]

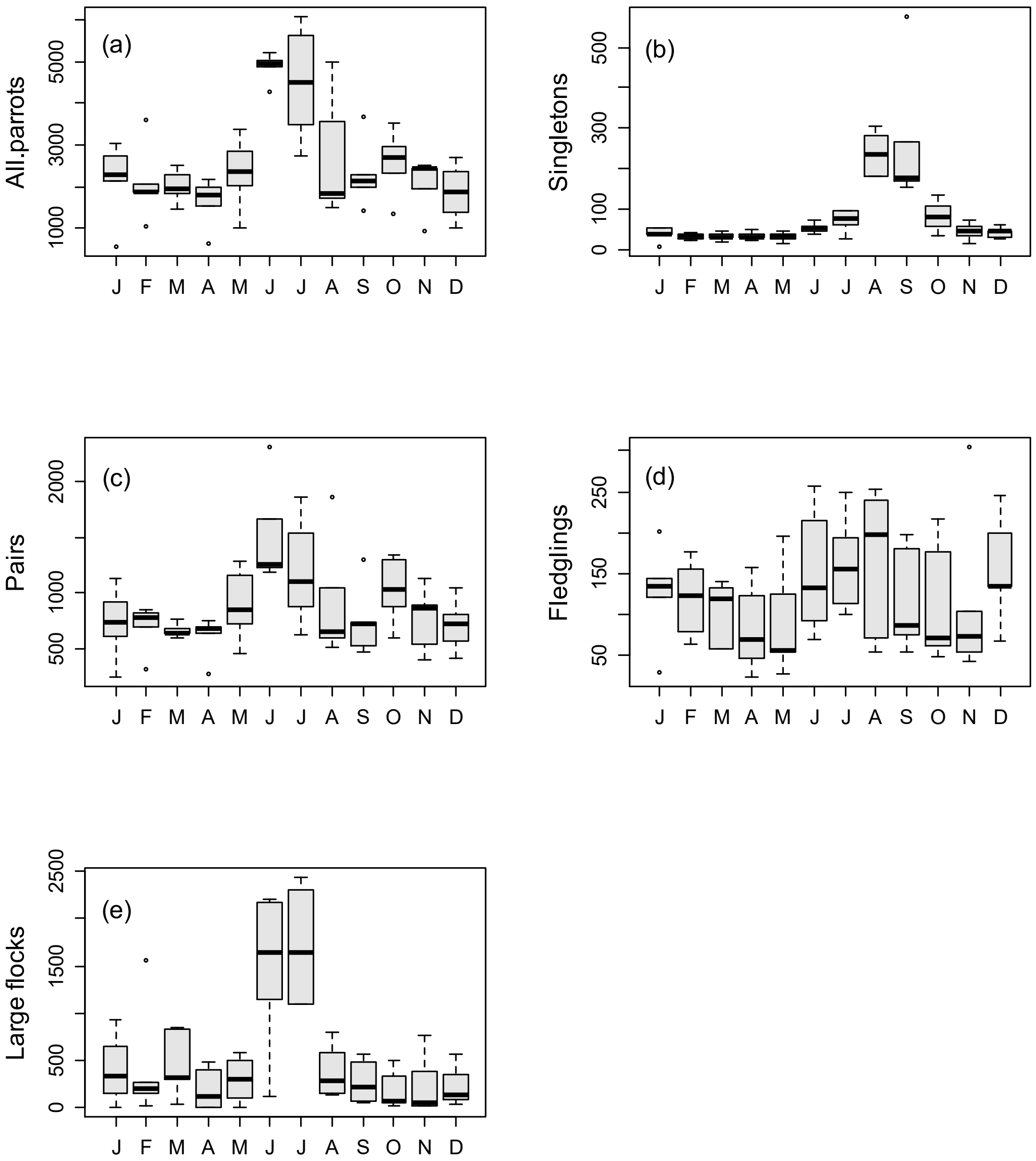

S1 Fig. Pooled counts of Blue-fronted Amazons in five roosts in the Pantanal of Brazil. (a) “All Parrots” refers to the numbers of parrots arriving the roosts irrespective of the flock size, (b) “Singletons” refers to the number of singletons, (c) “Pairs” refers to the number of pairs, (d) “Fledglings” refers to the number of fledglings parrots in family groups of three to six birds, and (e) “Large flocks” refers to parrots in flocks larger than six parrots. Counts were carried out from July 2004 to July 2009.
