# Supplementary material for: Communal roosts of the Blue-fronted Amazons (Amazona aestiva) in a large tropical wetland: Are they of different types?
Source: PLoS One. 2018 Oct 17;13(10):e0204824. doi: 10.1371/journal.pone.0204824 (PMC6192593; doi:10.1371/journal.pone.0204824)
Supplement: S11 Fig — (a) Monthly counts of parrots. (b) Seasonal pattern. (c) Seasonally adjusted trend of parrots along the study time. (d) The remainder. Counts were carried out from July 2004 to July 2009. (PDF) [file pone.0204824.s011.pdf]

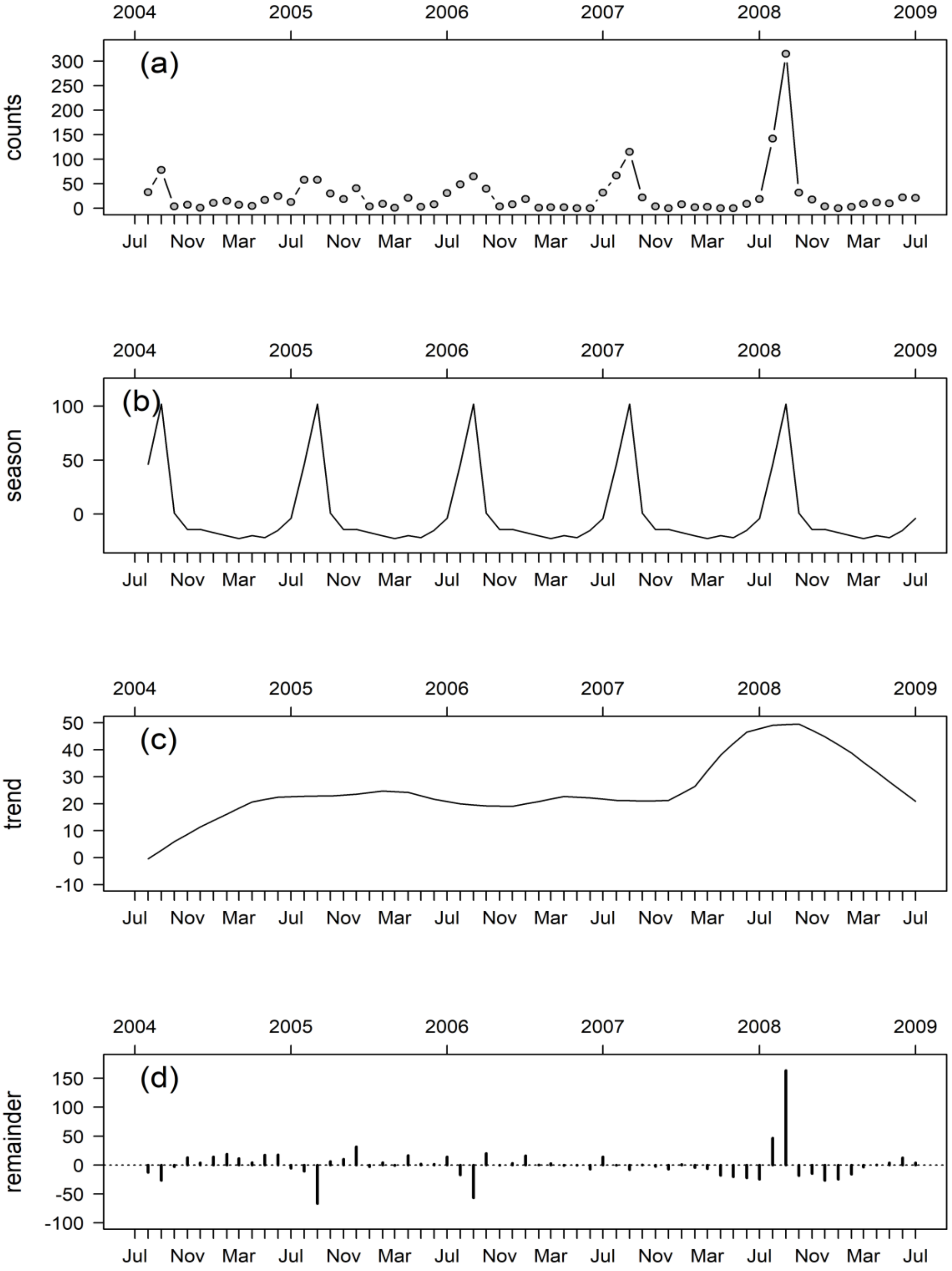

S11 Fig. Decomposition analysis of the monthly counts of the singletons of Blue-fronted Amazons in Roost 3 in the southern Pantanal of Brazil. (a) Monthly counts of parrots. (b) Seasonal pattern. (c) Seasonally adjusted trend of parrots along the study time. (d) The remainder. Counts were carried out from July 2004 to July 2009.
