# Supplementary material for: Communal roosts of the Blue-fronted Amazons (Amazona aestiva) in a large tropical wetland: Are they of different types?
Source: PLoS One. 2018 Oct 17;13(10):e0204824. doi: 10.1371/journal.pone.0204824 (PMC6192593; doi:10.1371/journal.pone.0204824)
Supplement: S20 Fig — (a) Monthly counts of pairs of parrots. (b) Seasonal pattern. (c) Seasonally adjusted trend of parrots along the study time. (d) The remainder. Counts were carried out from September 2004 to December 2006. (PDF) [file pone.0204824.s020.pdf]

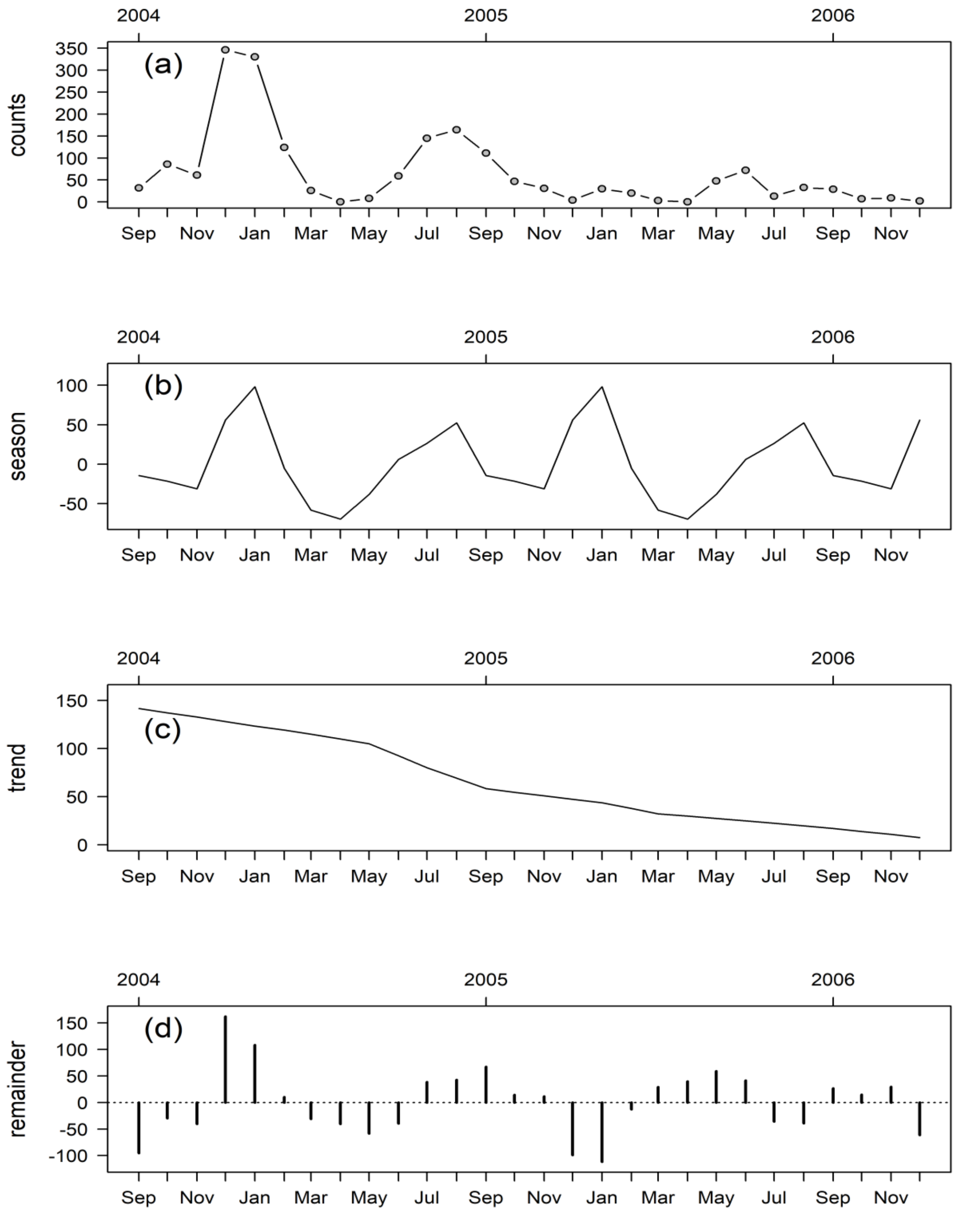

S20 Fig. Decomposition analysis of the monthly counts of the pairs of Blue-fronted Amazons in Roost 5 in the southern Pantanal of Brazil. (a) Monthly counts of pairs of parrots. (b) Seasonal pattern. (c) Seasonally adjusted trend of parrots along the study time. (d) The remainder. Counts were carried out from September 2004 to December 2006.
