# Supplementary material for: Communal roosts of the Blue-fronted Amazons (Amazona aestiva) in a large tropical wetland: Are they of different types?
Source: PLoS One. 2018 Oct 17;13(10):e0204824. doi: 10.1371/journal.pone.0204824 (PMC6192593; doi:10.1371/journal.pone.0204824)
Supplement: S22 Fig — Counts were carried out in four roosts in the Pantanal of Brazil from September 2004 to December 2006. Bands represent the 95% confidence interval. (PDF) [file pone.0204824.s022.pdf]

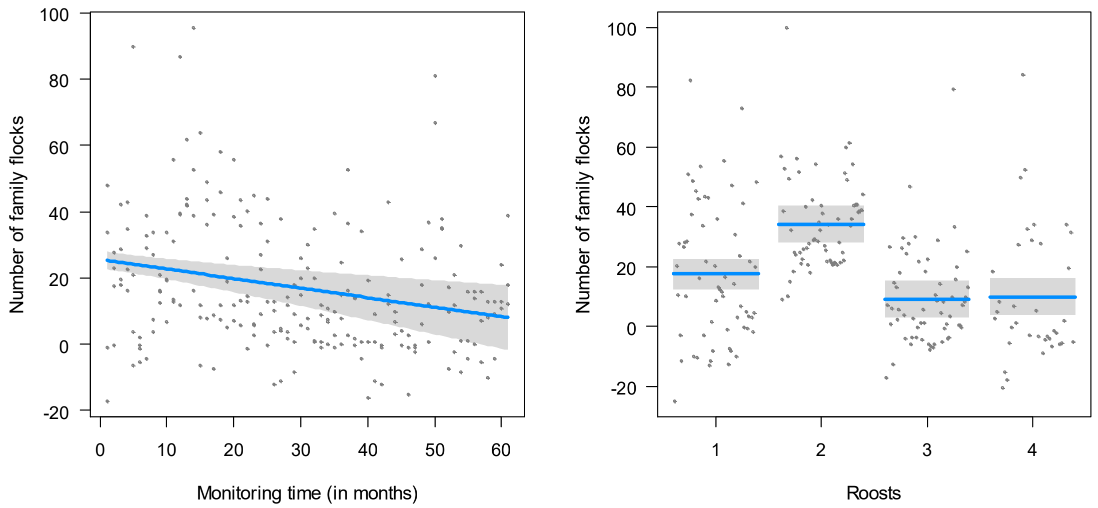

S22 Fig. Partial residuals of the permuted ANCOVA model relating the number of family flocks of Blue-fronted Amazons with (a) the monitoring time, and (b) roosts. Counts were carried out in four roosts in the Pantanal of Brazil from September 2004 to December 2006. Bands represent the 95% confidence interval.
