# Supplementary material for: Communal roosts of the Blue-fronted Amazons (Amazona aestiva) in a large tropical wetland: Are they of different types?
Source: PLoS One. 2018 Oct 17;13(10):e0204824. doi: 10.1371/journal.pone.0204824 (PMC6192593; doi:10.1371/journal.pone.0204824)
Supplement: S23 Fig — Counts were carried out in four roosts in the Pantanal of Brazil from September 2004 to December 2006. Bands represent the 95% confidence interval. (PDF) [file pone.0204824.s023.pdf]

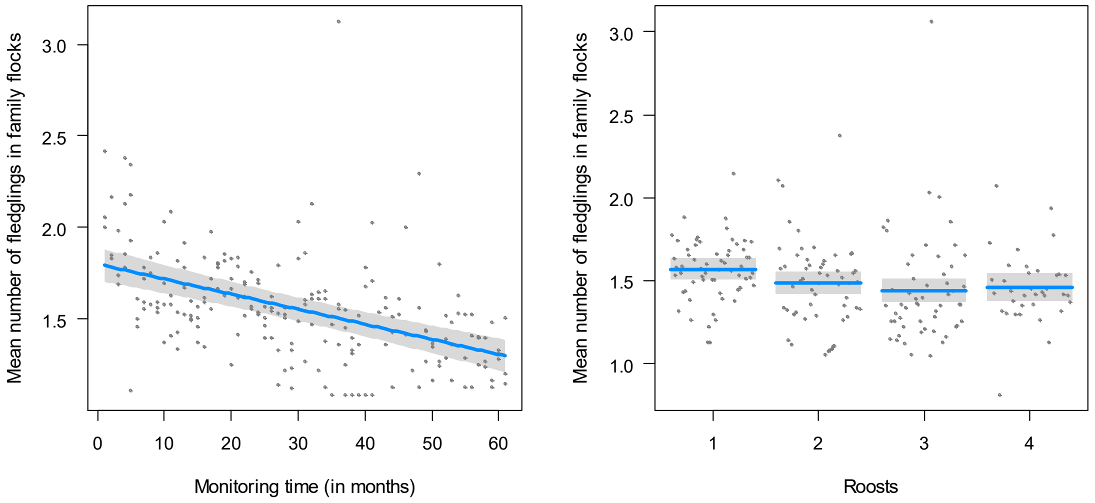

S23 Fig. Partials residuals of the ANCOVA model relating the mean number of fledglings of Blue-fronted Amazons in family groups with (a) the monitoring time, and (b) roosts. Counts were carried out in four roosts in the Pantanal of Brazil from September 2004 to December 2006. Bands represent the 95% confidence interval.
